# Which host-dependent insects are most prone to coextinction under changed climates?

**DOI:** 10.1002/ece3.1021

**Published:** 2014-03-17

**Authors:** Melinda L Moir, Lesley Hughes, Peter A Vesk, Mei Chen Leng

**Affiliations:** 1 School of Plant Biology, University of Western AustraliaCrawley, Western Australia, 6009, Australia; 2 School of Botany, University of MelbourneParkville, Victoria, 3010, Australia; 3 Department of Biological Sciences, Macquarie UniversityNorth Ryde, New South Wales, 2109, Australia

**Keywords:** Altitude, coextinction, extinction cascade, parasites, plant–insect interactions, secondary extinction, species loss, wetlands

## Abstract

Coextinction (loss of dependent species with their host or partner species) presents a threat to untold numbers of organisms. Climate change may act synergistically to accelerate rates of coextinction. In this review, we present the first synthesis of the available literature and propose a novel schematic diagram that can be used when assessing the potential risk climate change represents for dependent species. We highlight traits that may increase the susceptibility of insect species to coextinction induced by climate change, suggest the most influential host characteristics, and identify regions where climate change may have the greatest impact on dependent species. The aim of this review was to provide a platform for future research, directing efforts toward taxa and habitats at greatest risk of species loss through coextinction accelerated by climate change.

## Introduction

Anthropogenic changes to the environment including land clearing, pollution, introduced species, and climate change are precipitating a possible sixth mass extinction event (Warren et al. 2011; Bellard et al. 2012). Taxa that are dependent on specific host species comprise a large proportion of total biodiversity. Plant-dwelling insects, for example, are estimated to represent a quarter of all global terrestrial biodiversity (Strong et al. 1984). Although most groups are understudied, the number of species that we may lose from this component of diversity could be extremely large (Colwell et al. 2012). We have coined the term “cothreatened” to represent dependents that are at risk of extinction (Moir et al. 2011), and their extinction is termed “coextinction”, as it occurs either through the loss of the host or via a change in the host's population (Stork and Lyal 1993; Moir et al. 2010; Colwell et al. 2012).

Through the processes of altering seasonality, temperature, and rainfall, climate change may uncouple the relationships between hosts and dependent species, interfering with interactions essential for the survival of one or both species (Foden et al. 2008; Singer and Parmesan 2010; Kingsford and Watson 2011). For hosts alone, recent modeling demonstrates that climate change will reduce the population sizes of many plant species (e.g., Fitzpatrick et al. 2008; Mokany et al. 2012; Warren et al. 2013). Recently, Warren et al. (2013) assessed rates of loss for common plants under climate change and found 57% of species will lose more than half their current range by 2080. Species that already have small geographic ranges (not assessed by Warren et al. 2013 for reasons of data scarcity) are likely to be the most threatened by climate change (Thomas 2011), which suggests that a far higher proportion of the world's plant species are threatened with loss of at least half their ranges. Such decline in plant populations will undoubtedly affect plant-dwelling insects. Indeed, extinction rates for dependent species under altered climate change scenarios are predicted to be high (Thomas et al. 2006; Wilson and Maclean 2011). Furthermore, these predictions could be underestimates because they have been predominantly developed on a species-by-species basis, without considering coupled population dynamics and therefore the extra level of vulnerability associated with dependency.

Only about 30% of the 2.5–3.7 million insect species have been named (Hamilton et al. 2010). It is therefore difficult to assess the potential threat climate change presents for the majority of insects, particularly as current frameworks demand some background information on the target taxa (e.g., Thomas et al. 2011). In this article, we aimed to address this imbalance by reviewing the available literature and eliciting generalizations about the possible dependent insect groups at greatest risk from extinction through climate change. Using this literature, we identify the host plant traits that most likely influence dependent risk, and specific locations that could represent “hotspots” of coextinction via their exposure to particularly rapid or severe climate change. By identifying these taxa and habitats, we can begin to focus resources and implement climate change adaptation strategies to assist in the conservation and management of one of the largest components of the world's diversity, host-dependent species. Assessments of vulnerability to extinction have now been performed for many groups of species (i.e., Figure 4 in Bellard et al. 2012), but here, we focus specifically on how dependency adds another dimension to vulnerability. While the focus of the paper is on insect–plant interactions, many of the principles reviewed are also relevant to other types of host-dependent relationships, such as parasites and their hosts.

## Factors Increasing Propensity to Coextinction

A growing body of literature describes the traits that increase extinction risk (Purvis et al. 2000; Marini et al. 2012). Building on this, recent studies have characterized the traits that will disadvantage species and populations subject to a rapidly changing climate, and these can be broadly classified as follows: (1) specialized habitat or microhabitat requirements, (2) narrow environmental tolerances or thresholds, (3) dependence on environmental or specific cues/triggers that are disrupted by climate change, (4) dependence on interactions with particular species, (5) poor ability to disperse to or colonize suitable new habitats, and (6) small population size, area of occupancy or extent of occurrence (adapted from Foden et al. 2008; Thomas et al. 2011). For host-dependent species, the influence of these traits may be exacerbated due to the nature of the dependent's reliance on the survival and well-being of the populations of another species. For example, the critically endangered *Acizzia veski* is a herbivorous plant-louse that feeds only on the plant *Acacia veronica*, but *A. veronica* is restricted to gullies of one mountain range in south-western Australia (Taylor and Moir 2009). The plant-louse therefore has the first trait (1. specialized habitat), compounded by its obligate host also being a habitat specialist. Furthermore, the ecosystem in which the dependent insect and its host occur may be particularly vulnerable to climate change (Hughes 2011).

**Figure 1:**
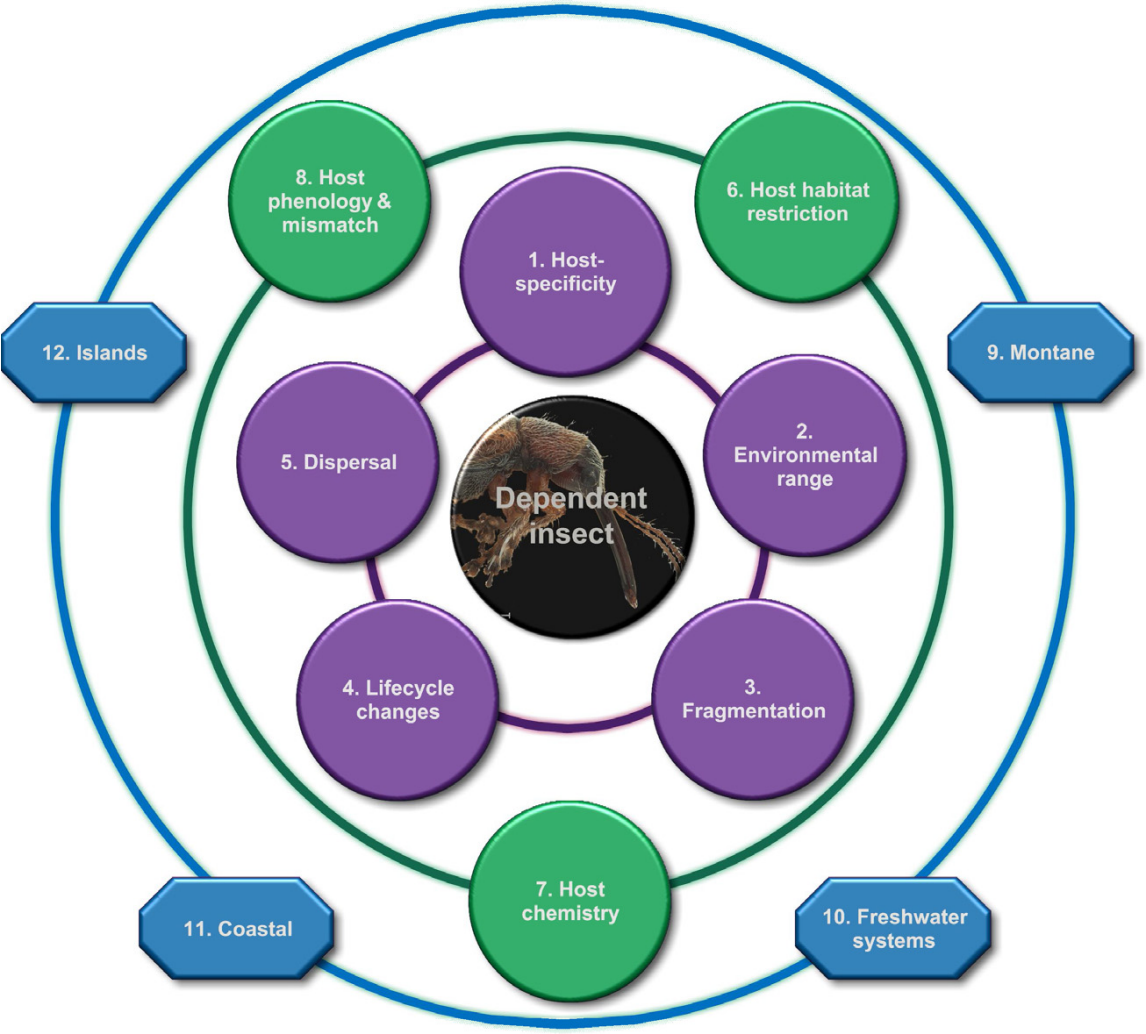
Circles of influence: the factors influencing the vulnerability of a herbivorous-dependent species to coextinction through climate change. Dependent traits are in purple in the inner circle, host factors are in green in the middle circle, and systems or locations are in blue in the outer circle.

A dependent insect species' propensity to be affected negatively by climate change is thus influenced by direct forces (dependent species traits), coupled with indirect forces (host factors and location). Figure 1 displays the traits that directly affect the dependent insect (in purple), those factors that affect the host species and therefore indirectly affect the dependent (in green), and those systems that predispose taxa in general toward negative impacts from a changing climate (in blue). These factors are given equal importance initially (Fig. 1), while in worked examples, an assessment of the relative importance of each factor is indicated by the factor's symbol varying in size in relation to the central insect button, and it is this assessment that is critical for subsequent conservation and management action.

The key factors from Fig. 1 are outlined below and are supplemented with a review of peer-reviewed journal papers on climate change and plant-dwelling insects. While numerous papers describe interactions between climate change and plant-dwelling insects, few include metrics for a viable meta-analysis of the traits most influential in determining extinction proneness. We recognize that our review reflects particular research interests, alongside successful publication, and may not necessarily translate directly to a particular trait or habitat being of greater biological importance in terms of risk. For this reason, we have included traits and habitats we believe are under-represented in current research efforts, such as islands and freshwater systems.

**Figure 2:**
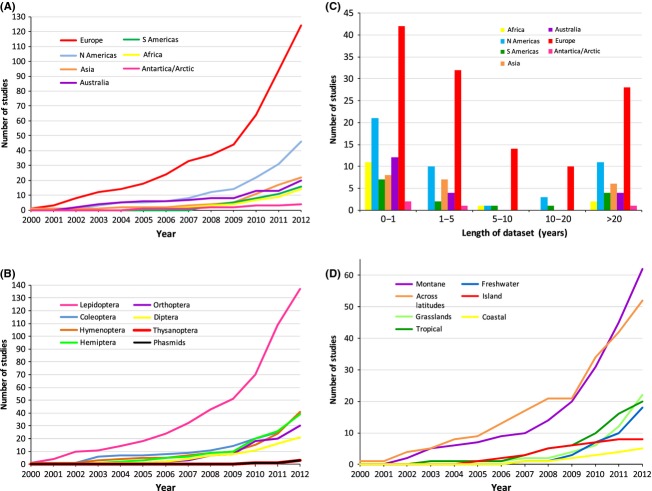
Cumulative number of studies gathered from the literature (within targets as defined in text) from 2000 to 2012 that address climate change and plant-dwelling insects (A) across different continents, (B) for each plant-dwelling insect order, (C) based on the length of the dataset and continent, and (D) for different locations.

The review encompasses papers published 2000–2012 inclusively, from Web of Science searches using the term *climate change*, with each of the following *butterfl*y, *moth, beetle*,*bug*,*stick insect*,*cricket*,*grasshopper*,*thrips*,*fly*,*bee*,*wasp*,*Lepidoptera*,*Hemiptera*,*Coleoptera*,*Orthoptera*,*Phasmatodea*,*Thysanoptera*,*Diptera*, and *Hymenoptera*. A 2000 review on climate change impacts (Hughes 2000) was a landmark from which studies were considered. Of a total 2014 papers, 236 were considered relevant and 1778 papers were excluded because they (1) were a review or meta-analysis, (2) focused predominantly on pest insects, and/or (3) did not include herbivorous or pollinator insect taxa (Fig. 2). Pest species were excluded because these are typically not native within the study region, are common, and are unlikely to suffer coextinction. For multiple studies on the same insect species, we condensed the information into a single record per species to avoid duplicating information (i.e., 29 papers became 13 records).

The review highlights that the impacts of climate change on plant-dwelling insects have been principally led by work in Europe and, to a lesser extent, North America (53% and 20% of studies, respectively; Fig. 2A). Under-represented continents were predominantly located in the Southern Hemisphere (Fig. 2A). Lepidoptera species have received the most attention (70% of studies; Fig. 2B), with a third of all studies conducted on Lepidoptera in Europe. Relatively few studies are available for other insect orders, particularly thrips (Thysanoptera) and stick insects (Phasmatodea; Fig. 2B). Studies utilizing short-term datasets (≤1 year) were predictably abundant (39% of studies), although unexpectedly, those based on long-term datasets (>20 years) represented almost a quarter of all studies (Fig. 2C). Finally, the predominant habitat for research into the impacts of climate change on insects was on mountains (65 papers), with all other habitats far less studied (Fig. 2D).

### Dependent: host specificity

**Figure 3:**
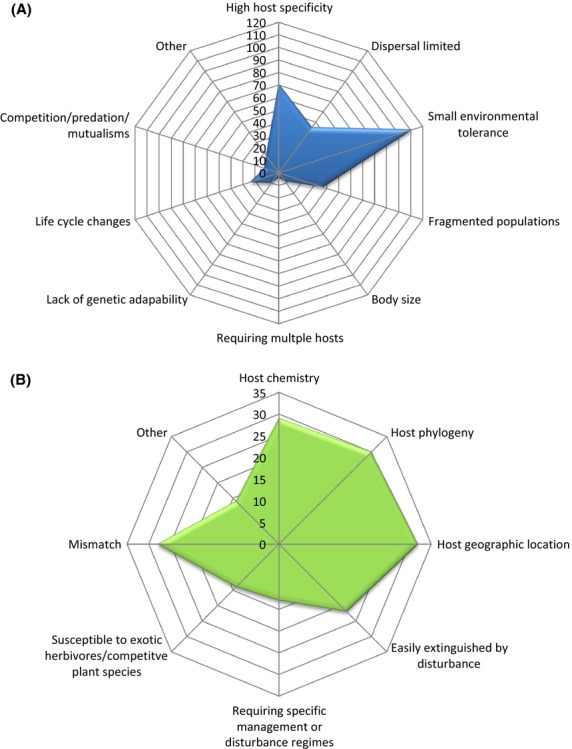
The number of published studies (2000–2012) that indicate those traits that increase plant-dwelling insect's propensity for extinction with climate change for (A) insect traits and (B) plant traits.

**Figure 4:**
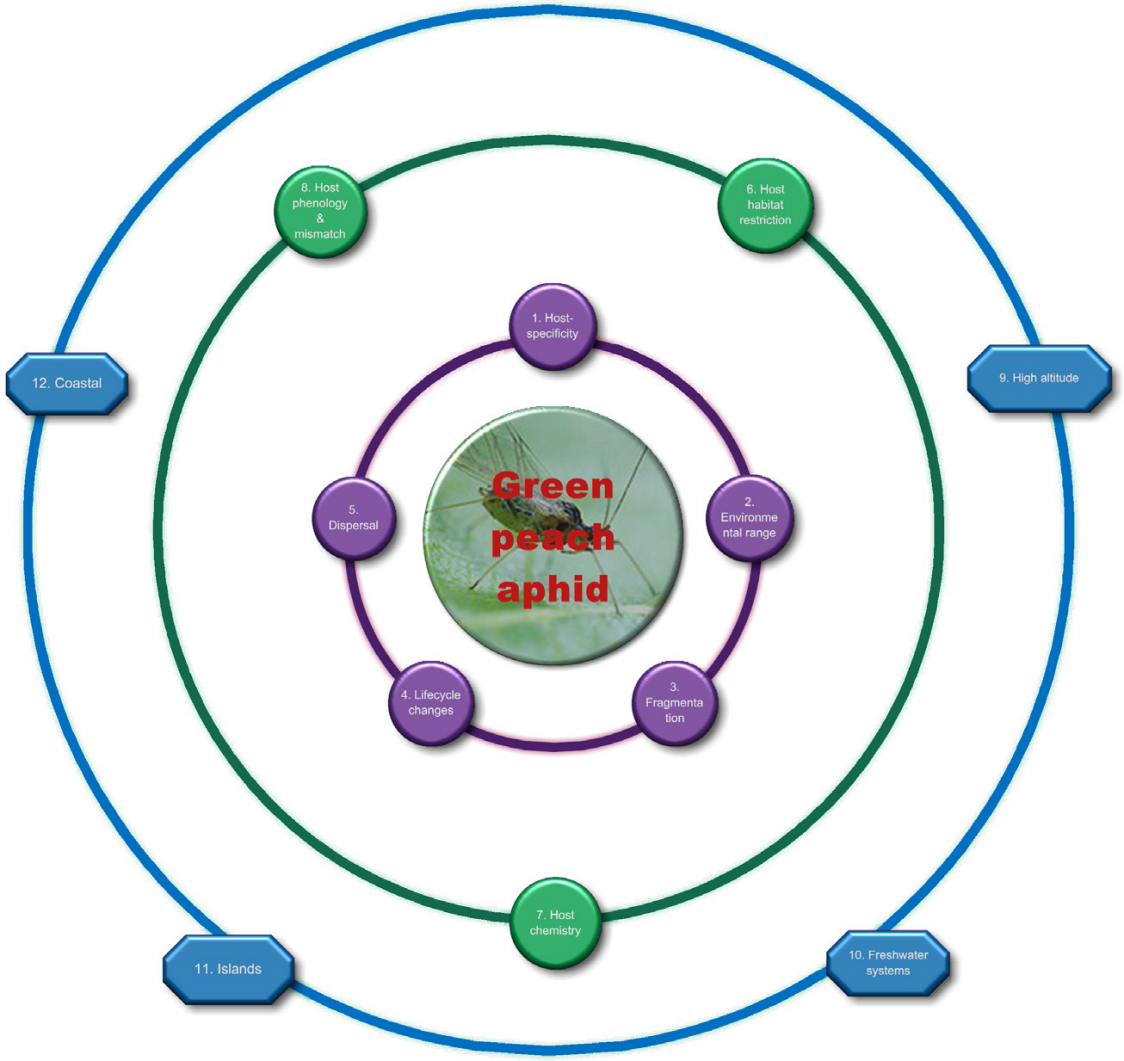
No circles of influence predicting the extinction of a herbivorous insect with climate change apply to the insect, the green peach aphid (*Myzus persicae*). This aphid occurs globally, has very broad environmental tolerances, except to cold conditions, has good powers of dispersal, and feeds from multiple plant families.

**Figure 5:**
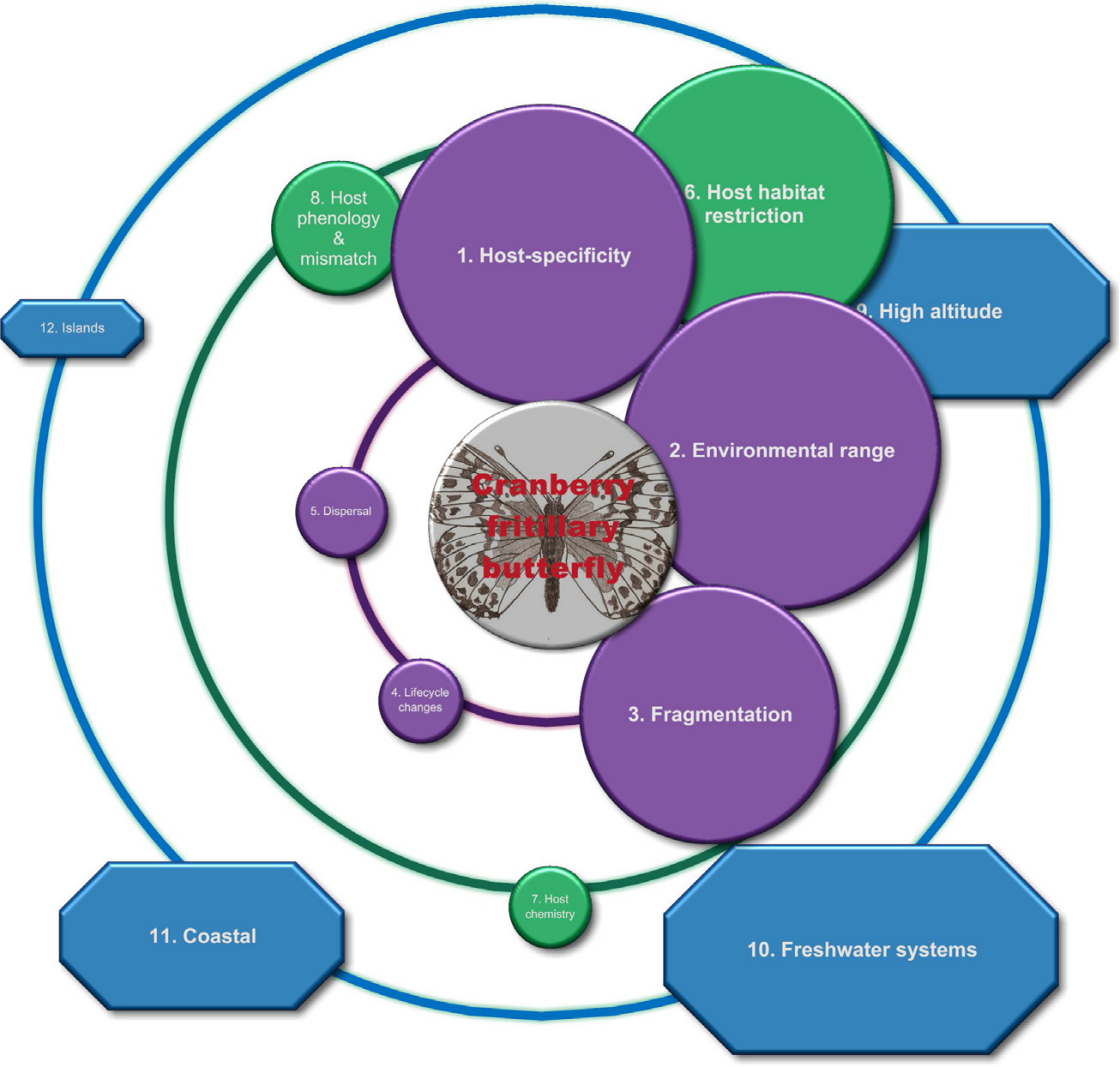
The circles of influence that determine the extinction of the Cranberry fritillary butterfly (*Boloria aquilonaris*) in Western Europe with climate change. The most influential are the host specificity of the butterfly to *Vaccinium oxycoccas* and its environmental tolerance, as well as the host's geographic location within peat bogs that either are close to the sea or on mountains (adapted from Schtickzelle et al. 2005).

High host specificity reduces the options of the dependent species for “jumping ship” onto other plant species if the host population declines or disappears (Moir et al. 2010; Colwell et al. 2012). Host specificity is considered highly influential on the vulnerability of dependent species to population decline or extinction (León-Cortés et al. 2003; Koh et al. 2004; Douda et al. 2012; Jönsson and Thor 2012), including under climate change (71 papers in our review considered higher host-specificity detrimental, Fig. 3A). For example, the Cranberry fritillary butterfly (*Boloria aquilonaris*: Fig. 5) is monophagous, feeding only on *Vaccinium oxycoccas*. Climate change may reduce the populations of host plants and thus will be highly influential in determining the butterfly's future survival (Schtickzelle et al. 2005). Some insects are so specialized that they require particular genetic populations of a host (e.g., *Boloria aquilonaris* on *Vaccinium oxycoccos*; Turlure et al. 2013), which may prevent dispersal to otherwise suitable habitats in a changing climate. In contrast, the low host specificity of the peach aphid means that this insect can feed on hosts from many different families, genera and species, and is unlikely to be threatened with extinction from climate change based on this trait alone (Fig. 4). In situations where climate change causes an increase in non-native plants, the likely result is a predominately generalist insect community, loss of many of the specialist insects, and ultimately, homogenization (=similarity) of insect assemblages in the region (e.g., De Sassi et al. 2012).

Host specificity can be even more restrictive in those dependent species that rely on multiple hosts during different stages of their life cycle (not to be confused with polyphagy; Koh et al. 2004). This is because the dependent must rely on the survival of multiple host species under climate change, as well as being at risk of phenological asynchrony with some or all hosts under altered environmental conditions (see Host: Phenology and mismatch below). Lycaenidae butterflies, and some leafhoppers and treehoppers (e.g., Pogonoscopini), provide examples of multiple host use, as they require both a host ant and host plant. The larvae of the Bathurst copper butterfly (*Paralucia spinifera*) feeds on only one variety of its host plant *Bursaria spinosa* subsp. *lasiophylla,* which occurs at higher altitudes (>900 m) compared with the more common form (Dexter and Kitching 1993). The butterfly also relies on the ant *Anonychomyrma itinerans*; the loss of this ant has been implicated in the butterfly's decline at one site (Dexter and Kitching 1993). Climate change has the potential to cause mismatches between the butterfly, plant, and ant. Of 77 Australian butterflies assessed at risk from climate change, Beaumont and Hughes (2002) noted that four of the seven high risk species were lycaenids requiring both ant and host plant. Although only 2% of studies in our review indicated that multiple host use is important (Fig. 3A), such a factor could greatly increase extinction risk through the factors represented in Fig. 1 being considered twice.

### Dependent: narrow environmental range

Temperature and water availability ultimately determine the environmental range to which the insect is limited. Climate change is predicted to increase temperatures in most terrestrial systems, particularly at low and mid-latitudes (IPCC 2013). It is not surprising, therefore, that narrow environmental tolerances were cited by the majority of papers as the greatest risk to insects under climate change (112 studies; Fig. A). Given the short-generation times of most insects, adaptation to new conditions can occur relatively quickly (e.g., Bradshaw et al. 2012). However, recent genetic work suggests that some insect traits, particularly environmental tolerances, cannot adapt rapidly to changing climatic conditions because such traits are linked to evolutionary conservative climate responses (Kellermann et al. 2012a,b).

**Figure 6:**
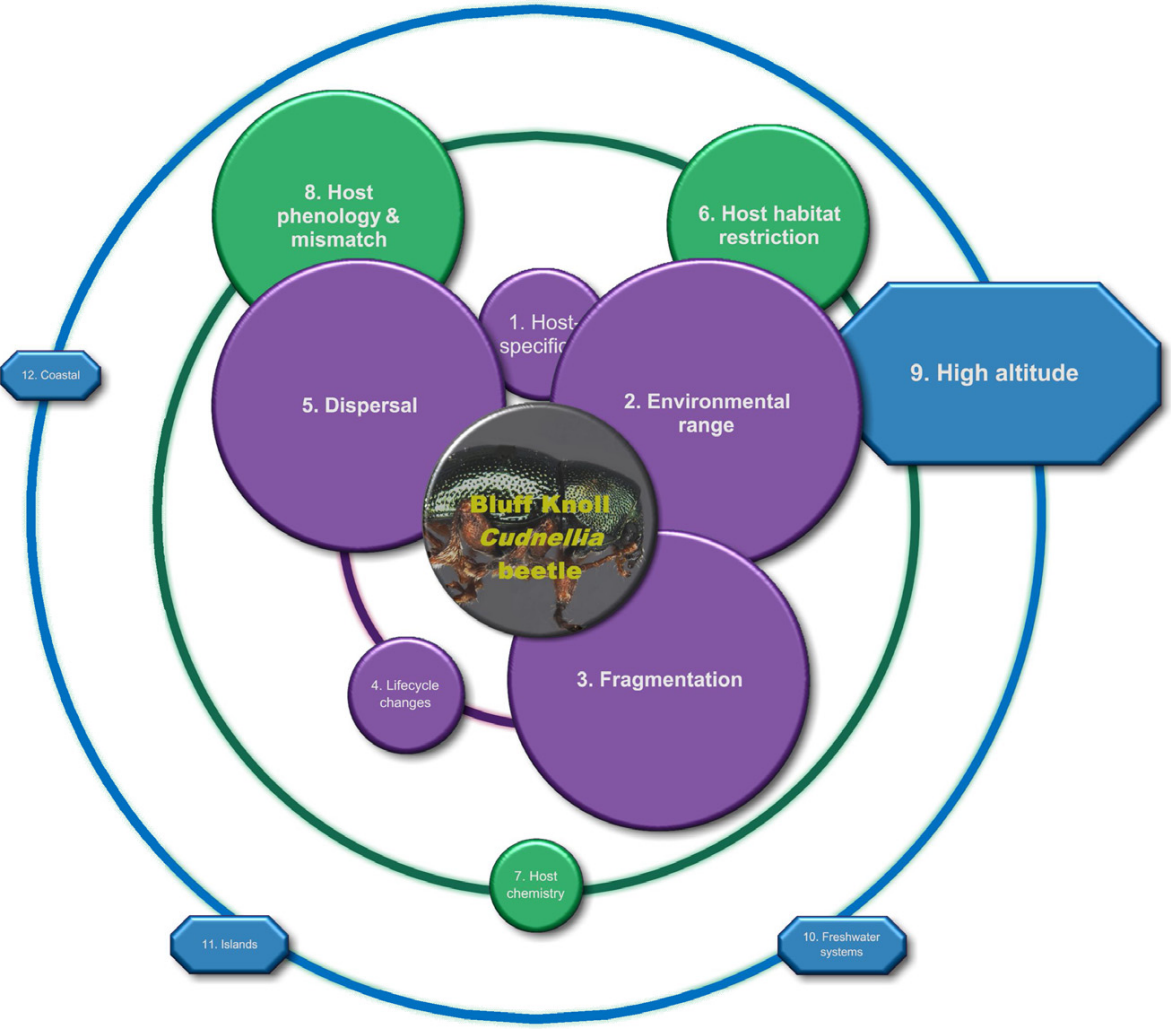
The circles of influence predicting the Bluff Knoll leaf beetle (*Cudnellia* sp. nov.; Chrysomelidae: Coleoptera) potential for coextinction with climate change. It occurs on Ericaceae plants (*Leucopogon*,*Sphenotoma,* and *Andersonia*), on the summits of two peaks in southwestern Australia (M. L. Moir, unpublished data). The beetle may be restricted to these montane habitats due to narrow environmental tolerances, and without hind wings, it has limited powers of dispersal. Therefore, the most influential factors are likely to be dispersal, the environmental tolerance of the beetle, and fragmentation of the beetle's populations across the mountain summits.

The potential vulnerability associated with a narrow environmental range is demonstrated by the Bluff Knoll leaf beetle (*Cudnellia* sp. nov.), which occurs exclusively at altitudes above 800 m in the southwest of Australia (Fig. 6). Explanatory variables for the current restricted distribution of the beetle (and a suite of other co-occurring invertebrates) include high humidity and relatively constant mild temperatures (Moir and Leng 2013). Climate change may reduce humidity and increase temperature variation, which will likely increase its risk of extinction under a changing climate (Moir and Leng 2013). Similarly, increasing temperature sets the lower altitudinal limit of 900 m for the black-veined white butterfly (*Aporia crataegi*) in mountains in Spain (Merrill et al. 2008). Local population extinctions of species with narrow environmental ranges have occurred, including the black-veined white butterfly and the cool-adapted Apollo butterfly (see further discussion below in Dependent: Fragmentation). Insect species that are restricted in range because they are adapted to cooler temperatures may be at particularly high risk of extinction because refugial zones will shrink disproportionately with climate change, compared with historical environmental fluctuations (Ohlemüller et al. 2008).

### Dependent: fragmentation

Here, we define fragmentation as the isolation of a dependent population from other populations of the same dependent species by natural (e.g., mountains or islands; see Location: Montane and Location: Islands) or anthropogenic barriers (e.g., land clearing). Dependent populations that are fragmented are often, but not always, associated with the fragmentation of host populations. At the most basic level, anthropogenically fragmented landscapes contain fewer plant species, herbivorous insects, and associated parasites than nonfragmented areas (Fenoglio et al. 2012). In naturally fragmented systems, host populations that have been separated for a long time may support genetically different insect populations (Borer et al. 2012) or different insect assemblages (Moir and Leng 2013). Fragmented landscapes can result in host populations that are too small to sustain viable populations of dependents, particularly specialist species (Piessens et al. 2009; Burkle and Knight 2012). Small and completely isolated populations are especially vulnerable to extinction through a single major disturbance (Wootton and Pfister 2013) or due to high genetic load (Mattila et al. 2011) and reduced fitness (Hanski 2013). This is termed an extinction vortex (*sensu* Gilpin and Soulé 1986); once a population reaches a critically small size, local extinction may be inevitable through demographic stochasticity, environmental stochasticity or genetic factors (Gilpin and Soulé 1986).

Climate change may fragment populations directly; for example, lower altitude species may move up mountains as the climate changes, until populations are no longer connected at low altitudes. As climate change is predicted to reduce the area of occupancy for many plants, including common and widespread species (Fitzpatrick et al. 2008; Ohlemüller et al. 2008; Mokany et al. 2012), the habitat available to dependent insects will subsequently be less. Such a reduction will be critically important in determining whether insect populations remain viable on these smaller host populations, especially if host populations were already fragmented. Additionally, climate change may act synergistically with other disturbances to exacerbate the effects of fragmentation and cause local population extinctions. For example, we propose that increasing fire frequency and intensity, predicted to occur with climate change (Brennan et al. 2009; Bradstock 2010), may remove above-ground host plant biomass and extinguish dependent populations without allowing time for recruitment. Loss of favorable habitat resulting in fragmentation of insect and/or host plant populations with climate change was cited as a risk to plant-dwelling insects in 46 of reviewed studies (Fig. 3A). Potentially, more studies could have assigned this factor as a threat, but the decline or risk was attributed to other factors such as dispersal, host location, small environmental tolerances, or high host specificity. As Wilson and Maclean (2011) note, most conservation-listed insect species will not be able to colonize regions that become climatically favorable in the future because they have very specialized habitat requirements and occur in habitats that are highly fragmented.

Butterflies provide some of the best examples of the impact of fragmentation and climate change on populations, as they were the most studied group of insects (Fig. 2B). Mountain butterflies such as France's Apollo butterfly (*Parnassius apollo*), Spain's Black-veined white butterfly and Europe's Cranberry fritillary butterfly (Fig. 5) have experienced well-documented metapopulation extinctions within the last 100 years. Populations of these three species are naturally fragmented by mountains and patchy suitable habitat, but climate change and anthropogenic changes in land use have been specifically identified as the factors resulting in the extinctions (Schtickzelle et al. 2005; Descimon et al. 2005; Parmesan 2006; Merrill et al. 2008; Dieker et al. 2013). Fragmentation and low numbers of host plants probably caused the global extinction of the Hawaiian mealybug *Clavicoccus erinaceus* (IUCN 2013). The sole host, *Abutilon sandwicense*, is critically endangered with only 12 isolated subpopulations remaining (IUCN 2013). Thousands of insect species are potentially threatened by the synergism between habitat fragmentation and climate change, with important implications for conservation management. However, determining which insect species are most vulnerable is difficult to foresee given the complexity of the interactions; examining species ecological traits and functional groups in more detail with respect to these synergistic impacts is required (Mantyka-Pringle et al. 2012).

### Dependent: lifecycle changes

Climate change is altering the lifecycles of insect herbivores in multiple ways (see reviews by DeLucia et al. 2012; Jamieson et al. 2012). Warming can increase the number of insect generations per year, alter an insect's strategy to overwinter (e.g., as larvae instead of pupa), and reduce developmental times (Bale et al. 2002; Zvereva and Kozlov 2006; Altermatt 2010). These shifts can be beneficial for some species, but detrimental changes also occur. Global warming has been linked to reduced survival of insects during development, particularly over-wintering survival (Anderson et al. 2008; Bale and Hayward 2010). Insect survival is likely to be further reduced if warming creates mismatches between developmental times and the seasons (Sgolastra et al. 2012). Warming may also create mismatches in host plant and insect lifecycles (see Host: Phenology and mismatch). Changes in atmospheric gases may also lower the nutritional value of plants and slow developmental times or reduce survival of larvae (see Host: Chemistry below).

A moderate percentage of studies in our review noted changes in insect herbivore life cycles with climate change (24 papers; Fig. 3A). The majority of these studies did not, however, explicitly associate life cycle changes with the potential extinction of insect herbivores. We predict that this factor could be a driver of population extinction, especially if occurring concurrently with other factors, such as limited tolerances for temperature changes (see Dependent: narrow environmental range). For example, the decline in the garden tiger moth with warmer temperatures in the United Kingdom is linked to a combination of narrow environmental tolerances, poor powers of dispersal, and reduced over-winter survival (Anderson et al. 2008).

### Dependent: dispersal

Species with greater dispersal capacity generally have lower extinction rates because they are less likely to be isolated, demographically and genetically, than less mobile species (Thomas 2000; Macdonald and Johnson 2001). Such species are also more capable of migrating when conditions within their habitat become unfavorable (Denno et al. 1996). For some host-dependent species, this factor is straightforward to assess, with dispersal potential often positively related to the presence and size of wings (e.g., León-Cortés et al. 2003; Burke et al. 2011; Stevens et al. 2012). Our review identified poor dispersal as a moderately important factor contributing toward the predicted extinction of insects under a changing climate (45 studies; Fig. 3A), especially if the insects were required to move polewards to reach favorable climates. As an example, the previously mentioned Bluff Knoll beetle (Fig. 6) is flightless and restricted to montane areas on the eastern massif of the Stirling Range in Western Australia. Mountains close-by provide suitable habitat and host plants, but there is no evidence that the beetle can reach these habitats and is therefore likely to experience deteriorating conditions in the next 70 years through climate change and contracting habitat. We note though that if the insect is highly host specific, then its dispersal ability is further constrained by the dispersal ability of its host plant species.

### Host: habitat restriction

We define habitat restriction as the host plant's area of occupancy being small, commonly less than <2000 km^2^ (restricted area of occupancy according to IUCN 2013), and restricted to specific areas due to specialized habitat requirements, water requirements, environmental tolerances, or reliance by the plant on certain disturbance regimes. Although interactions with life history and disturbance regime are important, a plant's distribution pattern can influence its survival with climate change (Keith et al. 2008). Consequently, insect species that depend solely on plants with small, restricted distributions are also restricted geographically, such as the Cranberry fritillary butterfly, which is restricted to a host plant that is a peat-bog specialist (Fig. 5). Of the various ways that hosts may exert influence over dependent insects, the plant's habitat specialization was the most reported in our review (32 studies; Fig. 3B). Half of these studies focused on plants restricted to mountains (15 studies). Refugial zones also occur in coastal regions, gullies, islands, and wetlands (Fig. 1), but relatively few studies noted the restricted nature of plant species within these areas (7 studies).

The majority of studies on geographically restricted plants we reviewed specifically noted the importance of host specificity for extinction risk in the insects (24 of 32 studies). An additional dilemma for restricted taxa is that this may complicate migration to more suitable climates under global warming. Indeed, half of the papers that assessed geographically restricted hosts also noted the dispersal limitations imposed on the insects (15 studies). Alternatively, climate change may simply reduce the relevant habitat. Montane cloud forest, for example, is expected to shrink and with it the associated flora and fauna (Rojas-Soto et al. 2012).

### Host: chemistry

Host chemistry is complex and is predicted to alter in multiple ways through climate change. This, in turn, may affect the host plant selection, life cycle, reproduction and/or mortality of plant-dwelling insects. Less moisture in the form of rain or humidity will decrease the water content of plant tissue and could be detrimental to the survival of herbivorous insects, especially during developmental stages (Gibbs et al. 2011). Higher levels of CO_2_ in the atmosphere mean that there is more CO_2_ available for photosynthesis and can result in an increase in the C/N ratio, thereby “diluting” nitrogen levels (Stiling and Cornelissen 2007). In general, herbivores react to the lower leaf nutrient levels by increasing consumption, decreasing growth rates, and exhibiting lower abundances and diversity (Stiling and Cornelissen 2007; Cornelissen 2011). Increased atmospheric CO_2_ is also likely to dilute levels of N-based plant defensive chemicals such as cyanoglycosides, but increase carbon-based defensive chemicals, such as phenols; these changes will have species-specific impacts (Stiling and Cornelissen 2007). For example, insects with high host specificity may have adaptations that allow them to tolerate increases in plant defensive compounds in their host plants and related species better than generalist insects, but responses will also differ between feeding guilds (i.e., chewers, suckers, gallers, etc; Ali and Agrawal 2012). Elevated levels of O_3_ (ozone) are predicted to decrease the abundance and diversity of all insects by decreasing plant growth, lowering leaf nutritional quality, and increasing plant defensive compounds such as tannins (Hillstrom and Lindroth 2008; Cornelissen 2011). Dependent insects may also encounter changes in nutrient content and defensive compounds in their host plants through extreme weather events. For example, although the extent of snow cover in the Arctic is decreasing (IPCC 2013), in some areas, the amount of snow fall will increase and plants under prolonged snow cover could have higher leaf nitrogen content, which encourages herbivory (Torp et al. 2010). Similarly, drought could increase herbivory from particular insect species due to lower chemical defensive compounds (Gutbrodt et al. 2011) and higher foliar nutrient content in plants (Jactel et al. 2012). Responses of insect herbivores will vary though as other factors can influence herbivory, such as competition and other interactions (e.g., between root and foliar herbivores: Tariq et al. 2013).

We found that although 29 papers (Fig. 3B) examined changes in plant chemistry, the effects on insects are difficult to generalize. Most studies (19) considered plant chemistry for only one herbivore species, while papers considering multiple species demonstrated contrasting results depending on the insect or host plant species examined (e.g., Gutbrodt et al. 2011; Couture and Lindroth 2012). In addition, the impacts of different atmospheric changes occurring in combination (such as CO_2_ and O_3_) may affect plant chemistry in opposing directions. For example, Couture and Lindroth (2012) found that elevated atmospheric O_3_ resulted in reduced foliar quality in aspen, which was subsequently detrimental to gypsy moth feeding on these trees. In contrast, elevated CO_2_ increased foliar quality, which offset reductions caused by O_3_, and consequently ameliorated the overall effects on gypsy moth. We note that such offsets are, however, rarely predictable and may vary in different regions, and for different plant and herbivore species. The net impacts of changes in plant chemistry in combination with changes in temperature, moisture availability, atmospheric gases, and extreme weather events remain largely under-studied (Cornelissen 2011).

### Host: phenology and mismatch

In addition to locating host populations as they move or decline with climate change, some insects must time their lifecycles to coincide with that of critical periods of the plant, called phenological synchrony. Climate change is predicted to alter the timing of plant life cycles, and insect dependents that do not respond flexibly to this change may be negatively affected (Willis et al. 2008; DeLucia et al. 2012).

The mismatch in host-dependent life cycles through climatic warming has already caused local extinctions of Edith's checkerspot butterfly (*Euphydryas editha*) populations in the United States (McLaughlin et al. 2002; Singer and Parmesan 2010). The role of phenological mismatch is attracting growing attention; while only 10 papers were published on this topic prior to 2010, 20 were published between 2010 and 2012. This is possibly because longer datasets are required to detect not only the occurrence of mismatch in the field but also the consequences; 23 of the 30 mismatch studies used datasets >5 years old. Overall, 28 studies in our review indicated that changes in host plant phenology are likely to represent increased risk for both pollinator and herbivorous insects (Fig. 3B).

### Location: montane

Insects residing at higher altitudes, such as the Cranberry fritillary butterfly (Fig. 5) and Bluff Knoll beetle (Fig. 6), are more thermally restricted and are likely to respond most sensitively to rising temperatures (Hodkinson 2005; Hoiss et al. 2013). Global warming is altering the climate of high altitudinal zones to such an extent that certain habitats may disappear completely (some alpine habitats -Williams et al. 2007; cloud forests -Ponce-Reyes et al. 2013). The importance of species loss within altitudinal zones is reflected by reviews of plant-dwelling insect taxa across entire countries (e.g., England -Thomas et al. 2004, 2011) or over altitudinal transects (e.g., Costa Rica - Colwell et al. 2008 Fig 3B), which demonstrate that higher altitudinal insects are often most at risk of extinction due to their inability to adapt to warmer temperatures and lack of suitable habitat to migrate to. In our review, montane areas received the most attention of any ‘Location’; 65 studies (Fig. 2D) examined the impacts of climate change on insects within montane systems, particularly in Europe (39 studies). More recent studies have also highlighted the threat posed to taxa in nontemperate mountainous regions (e.g., Chen et al. 2009; Pyrcz and Garlacz 2012).

To survive, plant-dwelling insects must move to either higher altitudes or higher latitudes to keep pace with a suitable climate, or remain and adapt to the new climate. Insects that are able to remain within their original montane habitat (and perhaps thrive; see Nash et al. 2013) may have additional stressors to cope with. These include competition and predation from lower altitudinal species expanding upward (Molina-Montenegro et al. 2009; Franzen and Ockinger 2012; Imbert et al. 2012; Hoiss et al. 2012, 2013) and changes in obligate mutualisms with other organisms besides plants (Prado et al. 2010). The majority of montane studies (44) from our review highlighted that limited environmental tolerances were important, suggesting that many insect species would not be able to remain *in situ* with global warming. Some butterflies and moths are migrating to higher altitudes at a rate of 1–7 m per year (Wilson et al. 2005; Forister et al. 2010; Chen et al. 2009, 2011), but these rates are far slower than the predicted warming rates of ∼80 m year^−1^ for mountainous regions (Loarie et al. 2009). Therefore, mountain insects at highest risk of extinction are likely to have narrow environmental tolerances and be unable to migrate. As an example, the European Alp beetle *Oreina gloriosa* is at high risk because it is cold adapted and unable to migrate upward due to a combination of poor dispersal powers and a lack of host plants (Borer et al. 2012).

Although ensuring short-term survival, upward migration will eventually lead to a reduction in the range of mountaintop species because there is simply less area at higher altitudes to support sufficiently sized populations of all the species migrating upwards (Wilson et al. 2005; Forero-Medina et al. 2011; Hoiss et al. 2012). Furthermore, insect and plant populations will become more fragmented, with 17 montane studies in our review indicating it as a threat for insects. Ultimately, there is a limit to how high species can migrate, and current climate change predictions will very likely result in the extinction of many species in present-day summit communities (Parmesan 2006; Forero-Medina et al. 2011; Thomas et al. 2011). An assessment of threatened plant species in Tanzania found a positive correlation with altitude (Yessoufou et al. 2012), indicating that montane regions already contain large percentages of threatened plants that would be at further risk from climate change. Restriction of plants to summits represents an important threat to plant-dwelling insects, as noted by 15 montane studies we reviewed. Higher altitudes may consequently have greater proportions of insects at immediate risk of extinction than lower altitudes.

### Location: freshwater systems

We restrict focus here to dependent herbivorous insects (with or without an aquatic phase in their life cycle) that are reliant on some form of freshwater system (i.e., bog, marsh, stream, lake, river, etc) including riparian zones. Few studies have addressed the impact that climate change will have on these systems and their associated herbivores (18 studies surveyed here; Fig. 2D). This is surprising because such systems are at very high risk of alteration through a changing climate (Finlayson et al. 2013; Hughes 2011; Bush et al. 2012), and many could potentially disappear altogether. Insect turnover in these systems is high (Bush et al. 2012), indicating long-term historical fragmentation and low dispersal capacity of the biota. Populations of the Cranberry fritillary butterfly (Fig. 5), for example, are highly fragmented because it is specific to a host plant that is a bog specialist (Schtickzelle et al. 2005), and the impact of climate change on the host's habitat is likely to be substantial. We predict further research will highlight many herbivorous insects at risk of extinction in these zones, incorporating both aquatic and terrestrial taxa, and their assessment is urgent.

### Location: coastal

Terrestrial coastal habitats are being affected by global warming through rising sea levels and more frequent extreme weather events, which are increasing coastal erosion (FitzGerald et al. 2008; Finlayson et al. 2013; Hughes 2011). For example, erosion rates of Alaskan coastlines from storm surges have increased from 6 m/year in 1955–1979 to 17 m/year in 2007–2009 (Arp et al. 2010). Coastal zones generally have cooler and wetter conditions than those experienced inland, often resulting in many endemic, short-range species, particularly invertebrates and plants (e.g., Fischer et al. 2009; Moir et al. 2009; González-Orozco et al. 2011). Current climatic zones of coastal regions may disappear with global warming, particularly at the poleward extremes of continents (Williams et al. 2007). If present-day coastal zones move inland due to rising sea levels and coastal erosion, then coastal taxa would need to establish populations in the new regions. Evidence of species shifts from mountains and across latitudinal zones suggest that it is the highly mobile taxa that are moving, and not plants or the majority of invertebrates (Hughes 2012). Unfortunately, the fragmented nature of the landscape around most coastal zones, and the poor dispersal capabilities of most plants, suggests that range shifts will not be possible for many coastal plant species (Fischer et al. 2009; Gavin 2009) and, consequently, their plant-dwelling insect faunas.

A wide range of taxa are moving polewards to follow suitable climatic envelopes (Hickling et al. 2006). Coastlines may therefore represent the last suitable habitat for many taxa, both species that are endemic to the coast, as well as species that migrate to these zones to escape from increased temperatures in inland habitats. For example, the northern brown argus (*Plebeius (Aricia) artaxerxes*) and Scotch argus (*Erebia aethiops*) butterflies are at high risk of extinction through climate change as they are currently distributed in northern United Kingdom, and once they reach coastal zones, they will not be able to expand northward (Thomas et al. 2011).

Given the attention to poleward migration in the literature, the often greater threat status of coastal biota due to urbanization pressure, and the high vulnerability of coastal regions to climate change, particularly erosion and sea-level rise (FitzGerald et al. 2008), the lack of research on insects in these systems is baffling. Only five studies identified this region of high concern for plant-dwelling insects, despite numerous papers indicating that migration polewards will end at coastal zones.

### Location: islands

Islands represent natural forms of habitat fragmentation, with immigration and emigration between populations only possible for insect biota able to colonize either actively (i.e., strong flyers, swimmers) or passively (i.e., on wind currents, flotsam, or other animals; e.g., Murakami and Hirao 2010). Endemic suites of species have evolved, adapted to the conditions and to interactions with other taxa on particular islands (e.g., Price 2004; Stuart et al. 2012; Weigelt and Kreft 2013). Climate change threatens islands on several fronts. Firstly, sea-level rises are predicted to inundate some low-lying islands, extinguishing the plants and their associated insects (Ross et al. 2009). For the islands that remain, the rising seas will reduce the terrestrial and freshwater habitats available for taxa (Sodhi et al. 2009; Ross et al. 2009). Secondly, as climate change warms islands, they could experience more exotic species invasions and lose their native insect and plant species (Shaw et al. 2010). Finally, extreme meteorological events, which are predicted to increase with climate change, are often more detrimental on islands, and single events can extinguish species or substantially alter habitat (Ross et al. 2009). Dependents may experience deleterious impacts either directly from such weather events or indirectly through synergisms with other disturbances (i.e., Sinclair and Chown 2005).

The risk to plant-dwelling taxa on islands from climate change has largely been overlooked in the literature, with only 6 papers incorporating plant-dwelling insects and climate change (Fig. 2D). The available studies vary widely in focus although 4 of the 6 indicate that host specificity will be an important contributing factor in insect species extinctions. Other evidence suggests that islands may have a preponderance of generalist dependent faunas, with examples including insect herbivores (Ribeiro et al. 2005) and parasitic wasps (Santos et al. 2011). However, there are many exceptions to this; highly host-specific plant-lice have radiated in the Canary Islands (Percy 2003), as have host-specific leafhoppers in the Hawaiian Islands (Bennett and O'Grady 2012). Regardless of specificity, dependent insect species on islands are more likely to experience extinction because the additional pressure of wide-scale anthropogenic habitat destruction lessens the ability of the insects to withstand environmental stochasticity (i.e., Brook et al. 2003; Triantis et al. 2010).

Surprisingly, dispersal ability and fragmented population factors do not feature strongly in papers from our review, but we suspect this is due to being understudied rather than a true reflection of the most influential traits. It is essential that more research be conducted on plant-dwelling insects on islands, because islands have higher rates of species extinction than continents (e.g., Brook et al. 2003; Triantis et al. 2010), and insects face high levels of threat from climate change, rising sea-level and introduced species (Gerlach 2008).

## Discussion

We have presented the first synthesis of the main factors likely to influence the coextinction risk of plant-dwelling insects in the face of climate change, that have been subject to research, and proposed a novel schematic diagram that can be used when assessing the potential risk climate change represents for dependent species. The factors commonly cited in the literature as most influential in directly affecting insect species are environmental tolerances, host specificity, dispersal capabilities, population fragmentation, and life cycle changes. The three most important factors indirectly affecting the insects, by exerting pressure on host plants during climate change, are likely to be habitat restrictions of plant populations**,** changes in plant chemistry, and mismatch in the timing of plant and insect life cycles. Due to combinations of these direct and indirect factors, we expect that the locations where the majority of imminent coextinctions will occur are on mountains, islands, along coast lines and in habitats associated with freshwater systems.

To date, the majority of insect species identified as being at high risk of extinction have occurred at higher altitudes (Fig. 2D), although this is more likely a location bias of studies, rather than indicative of a general trend. Similarly, because of the predominantly Northern Hemisphere bias of studies in our review (Fig. 2A), we did not consider the habitat of grasslands as a key ‘Location’, despite 21 studies associated with this system. In the future, we expect other, currently understudied, habitat types such as heathland, rocky outcrops, semi-arid woodlands, broadleaf tropical forests, and cool temperate rainforest will yield many taxa identified as being at high risk. It is readily apparent that generalizations emerging from climate change research require testing in other regions of the world to ensure that the findings are consistent across taxa and locations.

### Global hotspots for loss of species through coextinction

Many of the ‘Global 200’, which represent 238 ecoregions of exceptional diversity (Olson and Dinerstein 2002) and incorporate biodiversity hotspots (Myers et al. 2000), are likely to experience extreme climatic conditions with global warming this century (Beaumont et al. 2011). As these regions already support many range-restricted, endemic plant species, the number of plant extinctions facilitated by climate change is expected to be high (Thomas et al. 2004; Malcolm et al. 2006). For example, modeled effects of climate change on the speciose genus *Banksia* in the hotspot of southwestern Australia reveal that the majority of species could experience population declines, with some species at risk of extinction, in the next 100 years (Fitzpatrick et al. 2008; Yates et al. 2010). Regions of exceptional plant diversity could contain the highest richness of plant-dwelling insects precisely because of the high host diversity. For example, Fonseca (2009) estimated that biodiversity hotspots contain approximately 796,000–1,602,000 monophagous (or host specific) plant-dwelling insects in total.

We have identified four general locations from the literature for which rates of extinction are generally considered likely to increase for many taxa, including herbivorous insects, due to climate change (mountains, coastal zones, islands, freshwater systems). We expect that numbers of insect species lost to coextinction will be especially high when mountains, islands, coastal, and freshwater systems occur within biodiversity hotspots and the identified Global 200, predominantly due to the very high numbers of host plant species at risk. For example, Cameroonian highland forests (West Africa hotspot) incorporate mountains; south coast of Western Australia (southwest Australia hotspot) contains mountains, coastal zones, and wetlands; and the islands of Indonesia (Wallacea hotspot) contain island, montane, coastal, and freshwater systems. All of these regions are predicted to have 9+ months of extreme climatic conditions by 2070 (Beaumont et al. 2011, fig. 3), which may further exacerbate extinction rates when compared to regions elsewhere.

### Conserving dependents threatened with coextinction through climate change

The impact of climate change on dependent species is difficult to predict given the complexities of interactions between different climatic variables, uncertainty in species responses, and species interactions with one another (Berg et al. 2010; Cornelissen 2011). It is therefore challenging to predict, and subsequently mitigate, species extinctions. Focusing on the persistence of plants alone may be insufficient to maintain dependent insect species, because this management strategy does not comprehensively account for the factors that can influence the survival of insects, such as vegetation structure, phenological mismatch, minimal population sizes of host plant required to sustain viable insect populations, or competition from other herbivorous insects. Furthermore, conservation actions for hosts threatened by climate change may include assisted migration, botanical garden cultivation, and seed banks, and such *ex situ* methods may accelerate the loss of dependent species because insects are not considered (Moir et al. 2012).

Similarly, relying solely on migration to prevent insect extinctions is risky. The current fragmented state of landscapes, the diminishing amount of undegraded habitat, combined with the velocity of temperature change means that many species are unlikely to be able to migrate to suitable habitats (Loarie et al. 2009). Despite evidence that some herbivorous insects are migrating with climate (e.g., Wilson et al. 2005; Hickling et al. 2006; Raxworthy et al. 2008; Chen et al. 2009), other plant-dwelling insects with poor dispersal capabilities, specialized habitats, or high host specificity are not migrating (Mattila et al. 2011; Borer et al. 2012). Wilson and Maclean (2011) therefore argue that estimates of future distribution sizes for threatened organisms should be based on a “no-dispersal” scenario.

The most effective climate change adaptation strategies for both hosts and dependent insects, indeed for most systems where complex interactions between species occur, is conserving the current environment and restoring fragmented habitat that may provide corridors to refugial areas (Gillson et al. 2013). Some have advocated creating new habitat to prepare for the arrival of climate refugees (Hodgson et al. 2011; Thomas 2011), but for plant-dwelling insects, this would require knowledge of the potential migrating insect's identity and their subsequent host requirements. Furthermore, Mair et al. (2014) found that for British butterflies, habitat restoration and creation are ineffective for species with declining abundances. In addition to habitat restoration, therefore, conservation resources should be directed toward reducing other threats that are exacerbated by climate change, such as large wildfires, invasive species, and spread of disease. Ignoring these other threats that interact with climate change will result in underestimates of the risk of extinction (Brook et al. 2008). Finally, assisted colonization and *ex situ* conservation may be the only remaining option for species that are not able to migrate independently and are unable to adapt to the new climate in their current habitat (Thomas 2011 and see decision frameworks of Moir and Leng 2013; Shoo et al. 2013).

## References

[b1] Ali JG, Agrawal AA (2012). Specialist versus generalist insect herbivores and plant defense. Trends Plant Sci.

[b2] Altermatt F (2010). Climatic warming increases voltinism in European butterflies and moths. Proc. Biol. Sci.

[b3] Anderson SJ, Conrad KF, Gillman MP, Woiwod IP, Freeland JR (2008). Phenotypic changes and reduced genetic diversity have accompanied the rapid decline of the garden tiger moth *Arctia caja* in the UK. Ecol. Entomol.

[b4] Arp CD, Jones BM, Schmutz JA, Urban FE, Jorgenson MT (2010). Two mechanisms of aquatic and terrestrial habitat change along an Alaskan Arctic coastline. Polar Biol.

[b6] Bale JS, Hayward SAL (2010). Insect overwintering in a changing climate. J. Exp. Biol.

[b1000] Bale JS, Masters GJ, Hodkinson ID, Awmack C, Bezemer TM, Brown VK (2002). Herbivory in global climate change research: direct effects of rising temperature on insect herbivores. Glob. Change Biol.

[b8] Beaumont LJ, Hughes L (2002). Potential changes in the distributions of latitudinally restricted Australian butterfly species in response to climate change. Glob. Change Biol.

[b2000] Beaumont LJ, Pitman A, Perkins S, Zimmermann NE, Yoccoz NG, Thuiller W (2011). Impacts of climate change on the world's most exceptional ecoregions. Proc. Natl Acad. Sci. USA.

[b2011] Beaumont LJ, Pitman A, Perkins S, Zimmermann NE, Yoccoz NG, Thuiller W (2011). Impacts of climate change on the world's most exceptional ecoregions. PNAS.

[b9] Bellard C, Bertelsmeier C, Leadley P, Thuiller W, Courchamp F (2012). Impacts of climate change on the future of biodiversity. Ecol. Lett.

[b10] Bennett GM, O'Grady PM (2012). Host–plants shape insect diversity: phylogeny, origin, and species diversity of native Hawaiian leafhoppers (Cicadellidae: Nesophrosyne). Mol. Phylogenet. Evol.

[b3000] Berg MP, Kiers ET, Driessen G, Kooi M, van der Heijden BW, Kuenen F (2010). Adapt or disperse: understanding species persistence in a changing world. Glob. Change Biol.

[b11] Borer M, Arrigo N, Buerki S, Naisbit RE, Alvarez N (2012). Climate oscillations and species interactions: large-scale congruence but regional differences in the phylogeographic structures of an alpine plant and its monophagous insect. J. Biogeogr.

[b12] Bradshaw WE, Emerson KJ, Holzapfel CM (2012). Genetic correlations and the evolution of photoperiodic time measurement within a local population of the pitcher-plant mosquito, *Wyeomyia smithii*. Heredity.

[b13] Bradstock RA (2010). A biogeographic model of fire regimes in Australia: current and future implications. Glob. Ecol. Biogeogr.

[b14] Brennan KEC, Christie FJ, York A (2009). Global climate change and litter decomposition: more frequent fire slows decomposition and increases the functional importance of invertebrates. Glob. Change Biol.

[b4000] Brook BW, Sodhi NS, Ng PKL (2003). Catastrophic extinctions follow deforestation in Singapore. Nature.

[b15] Brook BW, Sodhi NS, Bradshaw CJA (2008). Synergies among extinction drivers under global change. Trends Ecol. Evol.

[b16] Burke RJ, Fitzsimmons JM, Kerr JT (2011). A mobility index for Canadian butterfly species based on naturalists' knowledge. Biodivers. Conserv.

[b17] Burkle LA, Knight TM (2012). Shifts in pollinator composition and behavior cause slow interaction accumulation with area in plant-pollinator networks. Ecology.

[b18] Bush A, Nipperess D, Turak E, Hughes L (2012). Determining vulnerability of stream communities to climate change at the landscape scale. Freshw. Biol.

[b19] Chen IC, Shiu HJ, Benedick S, Holloway JD, Chey VK, Barlow HS (2009). Elevation increases in moth assemblages over 42 years on a tropical mountain. Proc. Natl Acad. Sci. USA.

[b20] Chen IC, Hill JK, Ohlemuller R, Roy DB, Thomas CD (2011). Rapid range shifts of species associated with high levels of climate warming. Science.

[b22] Colwell RK, Brehm G, Cardelus CL, Gilman AC, Longino JT (2008). Global warming, elevational range shifts, and lowland biotic attrition in the wet tropics. Science.

[b23] Colwell RK, Dunn RR, Harris NC (2012). Coextinction and persistence of dependent species in a changing world. Annu. Rev. Ecol. Evol. Syst.

[b24] Cornelissen T (2011). Climate change and its effects on terrestrial insects and herbivory patterns. Neotrop. Entomol.

[b25] Couture JJ, Lindroth RL (2012). Atmospheric change alters performance of an invasive forest insect. Glob. Change Biol.

[b26] De Sassi C, Lewis OT, Tylianakis JM (2012). Plant-mediated and nonadditive effects of two global change drivers on an insect herbivore community. Ecology.

[b27] DeLucia EH, Nabity PD, Zavala JA, Berenbaum MR (2012). Climate change: resetting plant-insect interactions. Plant Physiol.

[b28] Denno RF, Roderick GK, Peterson MA, Huberty AF, Dobel HG, Eubanks MD (1996). Habitat persistence underlies intraspecific variation in the dispersal strategies of planthoppers. Ecol. Monogr.

[b29] Descimon H, Bachelard P, Boitier E, Pierrat V, Kühn E, Feldmann R, Thomas J, Settele J (2005). Decline and extinction of Parnassius apollo populations in France - continued.

[b31] Dexter E, Kitching R, New T (1993). The Bathurst Copper, *Paralucia spinifera* Edwards and Common. Conservation biology of Lycaenidae butterflies.

[b32] Dieker P, Drees C, Schmitt T, Assmann T (2013). Low genetic diversity of a high mountain burnet moth species in the Pyrenees. Conserv. Genet.

[b33] Douda K, Horký P, Bílý M (2012). Host limitation of the thick-shelled river mussel: identifying the threats to declining affiliate species. Anim. Conserv.

[b34] Fenoglio MS, Srivastava D, Valladares G, Cagnolo L, Salvo A (2012). Forest fragmentation reduces parasitism via species loss at multiple trophic levels. Ecology.

[b35] Finlayson C, Davis J, Gell P, Kingsford R, Parton K (2013). The status of wetlands and the predicted effects of global climate change: the situation in Australia. Aquat. Sci.

[b36] Fischer DT, Still CJ, Williams AP (2009). Significance of summer fog and overcast for drought stress and ecological functioning of coastal California endemic plant species. J. Biogeogr.

[b37] FitzGerald DM, Fenster MS, Argow BA, Buynevich IV (2008). Coastal impacts due to sea-level rise. Annu. Rev. Earth Planet. Sci.

[b38] Fitzpatrick MC, Gove AD, Sanders NJ, Dunn RR (2008). Climate change, plant migration, and range collapse in a global biodiversity hotspot: the Banksia (Proteaceae) of Western Australia. Glob. Change Biol.

[b39] Foden W, Mace GM, Vié J-C, Angulo A, Butchart SH, DeVantier L, Jean-Christophe V, Hilton-Taylor C, Stuart SN (2008). Species susceptibility to climate change impacts. Wildlife in a changing world–an analysis of the 2008 IUCN Red List of threatened species.

[b40] Fonseca CR (2009). The silent mass extinction of insect herbivores in biodiversity hotspots. Conserv. Biol.

[b41] Forero-Medina G, Joppa L, Pimm SL (2011). Constraints to species' elevational range shifts as climate changes. Conserv. Biol.

[b42] Forister ML, McCall AC, Sanders NJ, Fordyce JA, Thorne JH, O'Brien J (2010). Compounded effects of climate change and habitat alteration shift patterns of butterfly diversity. Proc. Natl Acad. Sci.

[b44] Franzen M, Ockinger E (2012). Climate-driven changes in pollinator assemblages during the last 60 years in an Arctic mountain region in Northern Scandinavia. J. Insect Conserv.

[b45] Gavin DG (2009). The coastal-disjunct mesic flora in the inland Pacific Northwest of USA and Canada: refugia, dispersal and disequilibrium. Divers. Distrib.

[b46] Gerlach J, New TR (2008). Preliminary conservation status and needs of an oceanic island fauna: the case of Seychelles insects. Insect conservation and Islands.

[b47] Gibbs M, Breuker H, Van Dyck CJ (2011). Development on drought stressed host plants affects life history, flight morphology and reproductive output relative to landscape structure. Evol. Appl.

[b48] Gillson L, Dawson TP, Jack S, McGeoch MA (2013). Accommodating climate change contingencies in conservation strategy. Trends Ecol. Evol.

[b49] Gilpin ME, Soulé ME, Soulé ME (1986). Minimum viable populations: The processes of species extinctions. Conservation biology: the science of scarcity and diversity.

[b50] González-Orozco CE, Laffan SW, Miller JT (2011). Spatial distribution of species richness and endemism of the genus Acacia in Australia. Aust. J. Bot.

[b51] Gutbrodt B, Mody K, Dorn S (2011). Drought changes plant chemistry and causes contrasting responses in lepidopteran herbivores. Oikos.

[b52] Hamilton AJ, Basset Y, Benke KK, Grimbacher PS, Miller SE, Novotný V (2010). Quantifying uncertainty in estimation of tropical arthropod species richness. Am. Nat.

[b53] Hanski I (2013). Extinction debt at different spatial scales. Anim. Conserv.

[b54] Hickling R, Roy DB, Hill JK, Fox R, Thomas CD (2006). The distributions of a wide range of taxonomic groups are expanding polewards. Glob. Change Biol.

[b55] Hillstrom ML, Lindroth RL (2008). Elevated atmospheric carbon dioxide and ozone alter forest insect abundance and community composition. Insect Conserv. Divers.

[b56] Hodgson JA, Thomas CD, Cinderby S, Cambridge H, Evans P (2011). Habitat re-creation strategies for promoting adaptation of species to climate change. Conserv. Lett.

[b57] Hodkinson ID (2005). Terrestrial insects along elevation gradients: species and community responses to altitude. Biol. Rev.

[b59] Hoiss B, Krauss J, Potts SG, Roberts S, Steffan-Dewenter I (2012). Altitude acts as an environmental filter on phylogenetic composition, traits and diversity in bee communities. Proc. Biol. Sci.

[b60] Hoiss B, Gaviria J, Leingärtner A, Krauss J, Steffan-Dewenter I (2013). Combined effects of climate and management on plant diversity and pollination type in alpine grasslands. Divers. Distrib.

[b61] Hughes L (2000). Global consequences of global warming: is the signal already apparent?. Trends Ecol. Evol.

[b62] Hughes L (2011). Climate change and Australia: key vulnerable regions. Reg. Environ. Change.

[b63] Hughes L, Lunney D, Hutchings P (2012). Can Australian biodiversity adapt to climate change?. Wildlife and climate change: towards robust conservation strategies for Australian fauna.

[b64] Imbert CE, Goussard F, Roques A (2012). Is the expansion of the pine processionary moth, due to global warming, impacting the endangered Spanish moon moth through an induced change in food quality?. Integr. Zool.

[b65] Stocker TF, Qin D, Plattner G-K, Tignor M, Allen SK, Boschung J, Nauels A, Xia Y, Bex V, Midgley PM, IPCC Summary for Policymakers. Climate Change 2013: The Physical Science Basis.

[b66] IUCN (2013). International Union for the Conservation of Nature IUCN red list of threatened species. Version 2013.1.

[b5000] Jactel H, Petit J, Desprez-Loustau ML, Delzon S, Piou D, Battisti A (2012). Drought effects on damage by forest insects and pathogens: a meta-analysis. Glob. Change Biol.

[b6000] Jamieson MA, Trowbridge AM, Raffa KF, Lindroth RL (2012). Consequences of climate warming and altered precipitation patterns for plant-insect and multitrophic interactions. Plant Physiol.

[b67] Jönsson MT, Thor G (2012). Estimating coextinction risks from epidemic tree death: affiliate lichen communities among diseased host tree populations of Fraxinus excelsior. PLoS ONE.

[b68] Keith DA, Akçakaya HR, Thuiller W, Midgley GF, Pearson RG, Phillips SJ (2008). Predicting extinction risks under climate change: coupling stochastic population models with dynamic bioclimatic habitat models. Biol. Lett.

[b69] Kellermann V, Overgaard J, Hoffmann AA, Fløjgaard C, Svenning J-C, Loeschcke V (2012a). Upper thermal limits of Drosophila are linked to species distributions and strongly constrained phylogenetically. Proc. Natl Acad. Sci.

[b70] Kellermann V, Loeschcke V, Hoffmann AA, Kristensen TN, Fløjgaard C, David JR (2012b). Phylogenetic constraints in key functional traits behind species' climate niches: patterns of desiccation and cold resistance across 95 drosophila species. Evolution.

[b71] Kingsford RT, Watson JE (2011). Climate change in Oceania - a synthesis of biodiversity impacts and adaptations. Pac. Conserv. Biol.

[b72] Koh LP, Dunn RR, Sodhi NS, Colwell RK, Proctor HC, Smith VS (2004). Species coextinctions and the biodiversity crisis. Science.

[b74] León-Cortés JL, Lennon JJ, Thomas CD (2003). Ecological dynamics of extinct species in empty habitat networks. 2. The role of host plant dynamics. Oikos.

[b75] Loarie SR, Duffy PB, Hamilton H, Asner GP, Field CB, Ackerly DD (2009). The velocity of climate change. Nature.

[b76] Macdonald DW, Johnson DD, Clobert J, Danchin E, Dhondt AA, Nichols JD (2001). Dispersal in theory and practice: consequences for conservation biology. Dispersal.

[b77] Mair L, Hill JK, Fox R, Botham M, Brereton T, Thomas CD (2014). Abundance changes and habitat availability drive species' responses to climate change. Nat. Clim. Change.

[b78] Malcolm JR, Liu C, Neilson RP, Hansen L, Hannah L (2006). Global warming and extinctions of endemic species from biodiversity hotspots. Conserv. Biol.

[b79] Mantyka-Pringle CS, Martin TG, Rhodes JR (2012). Interactions between climate and habitat loss effects on biodiversity: a systematic review and meta-analysis. Glob. Change Biol.

[b80] Marini L, Bruun HH, Heikkinen RK, Helm A, Honnay O, Krauss J (2012). Traits related to species persistence and dispersal explain changes in plant communities subjected to habitat loss. Divers. Distrib.

[b81] Mattila N, Kaitala V, Komonen A, Paivinen J, Kotiaho JS (2011). Ecological correlates of distribution change and range shift in butterflies. Insect Conserv. Divers.

[b82] McLaughlin JF, Hellmann JJ, Boggs CL, Ehrlich PR (2002). Climate change hastens population extinctions. Proc. Natl Acad. Sci.

[b83] Merrill RM, Gutierrez D, Lewis OT, Gutierrez J, Diez SB, Wilson RJ (2008). Combined effects of climate and biotic interactions on the elevational range of a phytophagous insect. J. Anim. Ecol.

[b84] Moir ML, Leng M-C (2013). Developing management strategies to combat increased coextinction rates of plant-dwelling insects through global climate change.

[b85] Moir ML, Brennan KEC, Harvey MS (2009). Diversity, endemism and species turnover of millipedes within the southwest Australia global biodiversity hotspot. J. Biogeogr.

[b86] Moir ML, Vesk PA, Brennan KE, Keith DA, Hughes L, McCarthy MA (2010). Current constraints and future directions in estimating coextinction. Conserv. Biol.

[b87] Moir ML, Vesk PA, Brennan KE, Keith DA, McCarthy LMA, Hughes L (2011). Identifying and managing cothreatened invertebrates through assessment of coextinction risk. Conserv. Biol.

[b88] Moir ML, Vesk PA, Brennan KE, Poulin R, Hughes L, Keith DA (2012). Considering extinction of dependent species during translocation, ex situ conservation, and assisted migration of threatened hosts. Conserv. Biol.

[b89] Mokany K, Harwood TD, Williams KJ, Ferrier S (2012). Dynamic macroecology and the future for biodiversity. Glob. Change Biol.

[b90] Molina-Montenegro MA, Briones R, Cavieres LA (2009). Does global warming induce segregation among alien and native beetle species in a mountain-top?. Ecol. Res.

[b91] Murakami M, Hirao T (2010). Nestedness of insect assemblages on small Bahamian islands: importance of spatial processes. Insect Conserv. Divers.

[b92] Myers N, Mittermeier RA, Mittermeier CG, Kent GA, Da Fonseca J (2000). Biodiversity hotspots for conservation priorities. Nature.

[b93] Nash MA, Griffin PC, Hoffmann AA (2013). Inconsistent responses of alpine arthropod communities to experimental warming and thermal gradients. Climate Res.

[b94] Ohlemüller R, Anderson BJ, Araújo MB, Butchart SH, Kudrna O, Ridgely RS (2008). The coincidence of climatic and species rarity: high risk to small-range species from climate change. Biol. Lett.

[b95] Olson DM, Dinerstein E (2002). The Global 200: priority ecoregions for global conservation. Ann. Mo. Bot. Gard.

[b96] Parmesan C (2006). Ecological and evolutionary responses to recent climate change. Annu. Rev. Ecol. Evol. Syst.

[b7000] Percy DM (2003). Radiation, diversity and host-plant interactions among island and continental legume-feeding psyllids. Evolution.

[b97] Piessens K, Adriaens D, Jacquemyn H, Honnay O (2009). Synergistic effects of an extreme weather event and habitat fragmentation on a specialised insect herbivore. Oecologia.

[b98] Ponce-Reyes R, Nicholson E, Baxter PW, Fuller RA, Possingham H (2013). Extinction risk in cloud forest fragments under climate change and habitat loss. Divers. Distrib.

[b99] Prado SS, Hung KY, Daugherty MP, Almeida RPP (2010). Indirect Effects of Temperature on Stink Bug Fitness, via Maintenance of Gut-Associated Symbionts. Appl. Environ. Microbiol.

[b100] Price JP (2004). Floristic biogeography of the Hawaiian Islands: influences of area, environment and paleogeography. J. Biogeogr.

[b101] Purvis A, Jones KE, Mace GM (2000). Extinction. BioEssays.

[b102] Pyrcz TW, Garlacz R (2012). The presence-absence situation and its impact on the assemblage structure and interspecific relations of Pronophilina Butterflies in the Venezuelan Andes (Lepidoptera: Nymphalidae). Neotrop. Entomol.

[b103] Raxworthy CJ, Pearson RG, Rabibisoa N, Rakotondrazafy AM, Ramanamanjato JB, Raselimanana AP (2008). Extinction vulnerability of tropical montane endemism from warming and upslope displacement: a preliminary appraisal for the highest massif in Madagascar. Glob. Change Biol.

[b8000] Ribeiro SP, Borges PAV, Gaspar C, Melo C, Serrano ARM, Amaral J (2005). Canopy insect herbivores in the Azorean Laurisilva forests: key host plant species in a highly generalist insect community. Ecography.

[b104] Rojas-Soto OR, Sosa V, Ornelas JF (2012). Forecasting cloud forest in eastern and southern Mexico: conservation insights under future climate change scenarios. Biodivers. Conserv.

[b105] Ross MS, O'Brien JJ, Ford RG, Zhang K, Morkill A (2009). Disturbance and the rising tide: the challenge of biodiversity management on low-island ecosystems. Front. Ecol. Environ.

[b9000] Santos AMC, Quicke DLJ, Borges PAV, Hortal J (2011). Species pool structure determines the level of generalism of island parasitoid faunas. J. Biogeogr.

[b106] Schtickzelle N, WallisDeVries MF, Baguette M (2005). Using surrogate data in population viability analysis: the case of the critically endangered cranberry fritillary butterfly. Oikos.

[b100000] Sgolastra F, Kemp WP, Maini S, Bosch J (2012). Duration of prepupal summer dormancy regulates synchronization of adult diapause with winter temperatures in bees of the genus Osmia. J. Insect Physiol.

[b107] Shaw JD, Spear D, Greve M, Chown SL (2010). Taxonomic homogenization and differentiation across Southern Ocean Islands differ among insects and vascular plants. J. Biogeogr.

[b10001] Shoo L, Hoffmann A, Garnett S, Pressey R, Williams Y, Taylor M (2013). Making decisions to conserve species under climate change. Clim. Change.

[b108] Sinclair BJ, Chown SL (2005). Deleterious effects of repeated cold exposure in a freeze-tolerant sub-Antarctic caterpillar. J. Exp. Biol.

[b109] Singer MC, Parmesan C (2010). Phenological asynchrony between herbivorous insects and their hosts: signal of climate change or pre-existing adaptive strategy?. Philos. Trans. R Soc. Lond. B Biol. Sci.

[b110] Sodhi NS, Brook BW, Bradshaw CJ, Levin SA (2009). Causes and consequences of species extinctions. The Princeton guide to ecology.

[b111] Stevens VM, Trochet A, Clobert H, Van Dyck J, Baguette M (2012). How is dispersal integrated in life histories: a quantitative analysis using butterflies. Ecol. Lett.

[b112] Stiling P, Cornelissen T (2007). How does elevated carbon dioxide (CO2) affect plant–herbivore interactions? A field experiment and meta-analysis of CO2-mediated changes on plant chemistry and herbivore performance. Glob. Change Biol.

[b113] Stork NE, Lyal CHC (1993). Extinction or ‘co-extinction’ rates. Nature.

[b114] Strong DR, Lawton JH, Southwood SR (1984). Insects on plants. Community patterns and mechanisms.

[b115] Stuart YE, Losos JB, Algar AC (2012). The island–mainland species turnover relationship. Proc. Biol. Sci.

[b116] Tariq M, Rossiter JT, Wright DJ, Staley JT (2013). Drought alters interactions between root and foliar herbivores. Oecologia.

[b117] Taylor GS, Moir ML (2009). In threat of co-extinction: two new species of Acizzia Heslop-Harrison (Hemiptera: Psyllidae) from vulnerable species of Acacia and Pultenaea. Zootaxa.

[b118] Thomas CD (2000). Dispersal and extinction in fragmented landscapes. Proc. Biol. Sci.

[b119] Thomas CD (2011). Translocation of species, climate change, and the end of trying to recreate past ecological communities. Trends Ecol. Evol.

[b120] Thomas CD, Cameron A, Green RE, Bakkenes M, Beaumont LJ, Collingham YC (2004). Extinction risk from climate change. Nature.

[b121] Thomas CD, Franco A, Hill JK (2006). Range retractions and extinction in the face of climate warming. Trends Ecol. Evol.

[b122] Thomas CD, Hill JK, Anderson BJ, Bailey S, Beale CM, Bradbury RB (2011). A framework for assessing threats and benefits to species responding to climate change. Methods Ecol. Evol.

[b123] Torp M, Olofsson J, Witzell J, Baxter R (2010). Snow-induced changes in dwarf birch chemistry increase moth larval growth rate and level of herbivory. Polar Biol.

[b124] Triantis KA, Borges PAV, Ladle RJ, Hortal J, Cardoso P, Gaspar C (2010). Extinction debt on oceanic islands. Ecography.

[b125] Turlure C, Radchuk V, Baguette M, Meijrink M, Vries A, den Burg MW (2013). Plant quality and local adaptation undermine relocation in a bog specialist butterfly. Ecol. Evol.

[b126] Warren R, Price J, Fischlin A, Midgley S, de la Nava Santos G (2011). Increasing impacts of climate change upon ecosystems with increasing global mean temperature rise. Clim. Change.

[b127] Warren R, VanDerWal J, Price J, Welbergen J, Atkinson I, Ramirez-Villegas J (2013). Quantifying the benefit of early climate change mitigation in avoiding biodiversity loss. Nat. Clim. Chang.

[b128] Weigelt P, Kreft H (2013). Quantifying island isolation–insights from global patterns of insular plant species richness. Ecography.

[b129] Williams JW, Jackson ST, Kutzbach JE (2007). Projected distributions of novel and disappearing climates by 2100 AD. Proc. Natl Acad. Sci.

[b130] Willis CG, Ruhfel B, Primack RB, Miller-Rushing AJ, Davis CC (2008). Phylogenetic patterns of species loss in Thoreau's woods are driven by climate change. Proc. Natl Acad. Sci.

[b131] Wilson RJ, Maclean IMD (2011). Recent evidence for the climate change threat to Lepidoptera and other insects. J. Insect Conserv.

[b132] Wilson RJ, Gutiérrez D, Gutiérrez J, Martínez D, Agudo R, Monserrat VJ (2005). Changes to the elevational limits and extent of species ranges associated with climate change. Ecol. Lett.

[b133] Wootton JY, Pfister CA (2013). Experimental separation of genetic and demographic factors on extinction risk in wild populations. Ecology.

[b134] Yates CJ, Elith J, Latimer AM, Midgley D, Le Maitre GF, Schurr FM (2010). Projecting climate change impacts on species distributions in megadiverse South African Cape and Southwest Australian Floristic Regions: opportunities and challenges. Austral Ecol.

[b135] Yessoufou K, Daru BH, Davies TJ (2012). Phylogenetic patterns of extinction risk in the Eastern Arc ecosystems, an African biodiversity hotspot. PLoS ONE.

[b12000] Zvereva EL, Kozlov MV (2006). Consequences of simultaneous elevation of carbon dioxide and temperature for plant-herbivore interactions: a metaanalysis. Glob. Change Biol.

